# Induction gemcitabine and cisplatin in locoregionally advanced nasopharyngeal carcinoma

**DOI:** 10.1186/s40880-019-0385-5

**Published:** 2019-06-25

**Authors:** Yuan Zhang, Ying Sun, Jun Ma

**Affiliations:** Department of Radiation Oncology, Sun Yat-sen University Cancer Center, State Key Laboratory of Oncology in South China, Collaborative Innovation Center for Cancer Medicine, Guangdong Key Laboratory of Nasopharyngeal Carcinoma Diagnosis and Therapy, Guangzhou, 510060 Guangdong P. R. China

**Keywords:** Gemcitabine, Cisplatin, Induction chemotherapy, Nasopharyngeal carcinoma

## Abstract

The standard of care for patients with locoregionally advanced nasopharyngeal carcinoma is concurrent platinum-based chemoradiotherapy. Existing literature have demonstrated that the addition of gemcitabine and cisplatin as induction chemotherapy in locoregionally advanced nasopharyngeal carcinoma may have promising efficacy but were from phase 2 clinical trials. Stronger evidence-based data in forms of phase 3 clinical trial investigating the survival benefits of adding gemcitabine and cisplatin induction chemotherapy for such patients have been urgently warranted. In one of our recent studies published in the *New England Journal of Medicine*, “Gemcitabine and cisplatin induction chemotherapy in nasopharyngeal carcinoma”, 480 locoregionally advanced nasopharyngeal carcinoma patients from 12 hospitals across China were randomly assigned in a 1:1 ratio to receive either chemoradiotherapy alone or gemcitabine plus cisplatin and chemoradiotherapy. Our findings evinced that, as compared to chemoradiotherapy alone, the addition of induction chemotherapy comprising of gemcitabine plus cisplatin to concurrent cisplatin-radiotherapy to patients with locoregionally advanced nasopharyngeal carcinoma was safe, demonstrated improved recurrence-free survival, overall survival, and distant recurrence-free survival, and marginally superior locoregional recurrence-free survival.

## Main text

Nasopharyngeal cancer is endemic in southern China [1]. It affected an estimated of 130,000 patients worldwide in 2018, and almost half of these cases were from China [2]. Nearly 70% of patients were diagnosed with locoregionally advanced disease at the time of presentation [3]. For these patients, concurrent chemoradiotherapy constitutes the backbone of treatment [4–6], and distant metastasis is the main cause of disease failure [7, 8]. Our previous phase 3 clinical study showed that induction treatment with docetaxel, cisplatin, and fluorouracil before concurrent chemoradiotherapy significantly improved failure-free survival and overall survival [9]. However, this triple-drug combo was found to be accompanied with significant adverse effects [9–11]. Based on the results of two phase 2 trials, induction gemcitabine and cisplatin was demonstrated to be effective in locoregionally advanced nasopharyngeal carcinoma with favorable safety profiles [12, 13]. However, whether the addition of induction gemcitabine and cisplatin to concurrent chemoradiotherapy could further improve the survival of these patients remained unclear. We, therefore, conducted a multicenter, randomized controlled phase 3 clinical trial to investigate the efficacy and safety profile of adding induction gemcitabine and cisplatin to concurrent chemoradiotherapy in locoregionally advanced nasopharyngeal carcinoma patients, which was recently published in the *New England Journal of Medicine*, entitled “Gemcitabine and cisplatin induction chemotherapy in nasopharyngeal carcinoma” [14].

The study was an open-label, parallel group, randomized phase 3 trial which enrolled patients from 12 hospitals across China. Eligibility criteria were age between 18 and 64 years; histological confirmation of non-keratinizing nasopharyngeal carcinoma; non-distant metastatic, newly diagnosed stage III–IVB (excluding T3–4N0) disease staged using the American Joint Committee on Cancer 7th edition/Union for International Cancer Control stage classification system); Karnofsky performance status (KPS) scores of at least 70, and adequate hematologic, renal and hepatic function. Key exclusion criteria were lactating or pregnant patients; treatment for palliative intent; a past history of malignancy; previous treatment [i.e. radiotherapy, chemotherapy, or surgery (except diagnostic procedures)] to the nasopharynx or neck; or other severe comorbid diseases.

All enrolled patients were randomized to receive induction gemcitabine and cisplatin plus concurrent chemoradiotherapy or concurrent chemoradiotherapy alone. Induction gemcitabine and cisplatin were given intravenously as gemcitabine 1 g/m^2^ on days 1 and 8, and cisplatin 80 mg/m^2^ on day 1; induction chemotherapy was administered once every 3 weeks for 3 cycles. Concurrent cisplatin with radiotherapy was administered intravenously at a dose of 100 mg/m^2^ every 3 weeks on days 1, 22, and 43. Intensity-modulated radiotherapy was mandatory in both groups. The primary endpoint was recurrence-free survival. Second endpoints included overall survival, locoregional recurrence-free survival, distant recurrence-free survival, adherence to treatment and treatment-related adverse events.

From 2013 to 2016, a total of 480 patients were enrolled in this study, of whom 242 patients were randomized to receive induction gemcitabine and cisplatin plus concurrent chemoradiotherapy (induction gemcitabine and cisplatin arm), and 238 to receive concurrent chemoradiotherapy alone (concurrent chemoradiotherapy arm). With a median follow-up of 42.7 months (range 3.5 to 65.0 months), patients in the induction gemcitabine and cisplatin arm had significantly higher 3-year recurrence-free survival (85.3% vs. 76.5%, stratified hazard ratio [HR], 0.51; 95% confidence interval [CI] 0.34 to 0.77; *P* = 0.001) and 3-year overall survival (94.6% vs. 90.3%, stratified HR, 0.43; 95% CI 0.24 to 0.77) compared with patients in the concurrent chemoradiotherapy arm. Additional gemcitabine and cisplatin also improved the distant recurrence-free survival (91.1% vs. 84.4%, stratified HR, 0.43; 95% CI 0.25 to 0.73) of the patients in the induction gemcitabine and cisplatin arm but demonstrated similar locoregional recurrence-free survival (91.8% vs. 91.0%, stratified HR, 0.77; 95% CI 0.42 to 1.41) as to the concurrent chemoradiotherapy arm.

Our exploratory analyses included per-protocol analysis and subgroup analysis. The per protocol population comprised of cases that received 3 cycles of induction gemcitabine and cisplatin and 2–3 cycles of concurrent cisplatin plus radiotherapy in the induction gemcitabine and cisplatin arm and those receiving 3 cycles of concurrent cisplatin plus radiotherapy in the concurrent chemoradiotherapy arm. The clinical advantage of induction gemcitabine and cisplatin was also evident when analyzed by the per-protocol population, with higher 3-year recurrence-free survival (85.5% vs. 77.8%, HR, 0.50; 95% CI 0.32 to 0.76) and overall survival (95.5% vs. 90.6%, HR, 0.44; 95% CI 0.23 to 0.84) observed in patients receiving induction gemcitabine and cisplatin.

Post hoc exploratory analysis was performed to assess the possible differential efficacy of induction gemcitabine and cisplatin in a range of baseline subgroups. The addition of induction gemcitabine and cisplatin was associated with a trend towards improved recurrence-free survival in all subgroups. Statistically significant improvement in recurrence-free survival was achieved in male patients (HR, 0.50; 95% CI 0.31 to 0.80), patients aged < 45 years (HR, 0.47; 95% CI 0.25 to 0.89), and those with Karnofsky scores of 90–100 (HR, 0.41; 95% CI 0.25 to 0.66), T4 stage disease (HR, 0.47; 95% CI 0.27 to 0.81), N2 category (HR, 0.25; 95% CI 0.13 to 0.49), or stage IVA (HR, 0.51; 95% CI 0.29 to 0.89).

Overall, the addition of induction gemcitabine and cisplatin to concurrent chemoradiotherapy significantly improved the recurrence-free survival and overall survival in the intention-to-treat and per-protocol analyses. Subgroup analyses further evinced that induction gemcitabine and cisplatin could have better efficacy for patients with T4 or N2 disease. However, these subgroup analyses were post hoc and should only be considered hypothesis-generating. Moreover, we emphasized that findings from this study were from patients meeting specified inclusion and those with exclusion criteria such as patients with keratinizing subtype nasopharyngeal carcinoma, aged ≥ 65 years old, or having severe comorbidities were excluded. Whether this induction regimen comprising of gemcitabine and cisplatin could globally improve the survival benefit to these patients needs further investigations, and thorough assessment of the patients’ characteristics and disease conditions are to be meticulously performed in the clinic before prescribing this regimen.

Regarding adherence to treatment, induction gemcitabine and cisplatin was well tolerated. 96.7% of patients completed the protocol-defined 3 cycles of induction chemotherapy. During the concurrent phase, 79.9% of patients in the induction gemcitabine and cisplatin arm and 95.8% of patients in the concurrent chemoradiotherapy arm received at least 200 mg/m^2^ of concurrent cisplatin. All patients in the induction gemcitabine and cisplatin arm completed radiotherapy, while 2 of the 237 patients in the concurrent chemoradiotherapy arm did not, because they declined to participate. High compliance rate to induction gemcitabine and cisplatin plus concurrent chemoradiotherapy provided the basis for its efficacy in nasopharyngeal carcinoma.

Concerning the treatment-related adverse events, severe late complications due to treatment were not increased with induction gemcitabine and cisplatin, although there was an increase in acute adverse events. During induction chemotherapy, 33.5% and 5.4% of patients experienced grade 3 and grade 4 adverse events, respectively. Neutropenia was the most common (49 patients [20.5%]), followed by leucopenia (26 [10.9%]), and vomiting (26 [10.9%]). During the entire treatment course, 181 patients (75.7%) in the induction gemcitabine and cisplatin arm and 132 (55.7%) in the concurrent chemoradiotherapy arm reported grade 3 or 4 adverse events. The induction chemotherapy group demonstrated a higher incidence of grade 3 or 4 neutropenia (67 patients [28.0%] vs. 25 [10.5%]), thrombocytopenia (27 [11.3%] vs. 3 [1.3%]), anemia (23 [9.6%] vs. 2 [0.8%]), nausea (55 [23.0%] vs. 33 [13.9%]), and vomiting (54 [22.6%] vs. 33 [13.9%]) as compared to the concurrent chemoradiotherapy arm. The overall incidences of toxicities were raised when giving induction gemcitabine and cisplatin. However, the major increased acute adverse events in this trial were hematological, including neutropenia, thrombocytopenia, and anemia, which were largely asymptomatic. Thus, the induction chemotherapy and concurrent chemoradiotherapy were well-tolerated despite the increased risk of adverse events.

Innovative approaches to tackle the NPC epidemic is warranted [15] and the next milestone in the treatment of NPC patients could be relying on the implementation of immune therapy [16]. Given the high efficacy and favorable safety profile of gemcitabine and cisplatin regimen, currently, several ongoing randomized trials (NCT03427827 and NCT03700476) are underway to evaluate whether the addition of immune checkpoint inhibitors to the backbone induction gemcitabine and cisplatin plus concurrent chemoradiotherapy regimen could provide further survival benefits in patients with NPC, which could represent a promising combination strategy.

## Conclusions

Our data demonstrated that the addition of induction chemotherapy with gemcitabine plus cisplatin to concurrent cisplatin-radiotherapy significantly improved recurrence-free survival among patients with locoregionally advanced nasopharyngeal carcinoma. Taken together with the well-tolerated toxicity profile and high compliance rates, induction gemcitabine and cisplatin plus concurrent chemoradiotherapy can be considered as a first-line treatment option in nasopharyngeal carcinoma patients with node-positive T3–4 and N2–3 disease.

## Data Availability

Not applicable.
